# Drunk or Dying? A Common Conclusion on Insufficient Evidence

**Published:** 1918-09-28

**Authors:** 


### THE EDITOR'S LETTER-BOX (*continued*).

### Drunk or Dying? A Common Conclusion on Insufficient Evidence.

*To the Editor of* THE HOSPITAL.

SIR,—Is it not time that coroners and juries made some stricture on the action of doctors in concluding that because a comatose patient's breath has the odour of alcohol he is certainly suffering from alcoholic coma, and relegating him cursorily to the police cell, when he should be in a hospital receiving the medical and surgical aid of which he is often in most urgent need if his life is to be saved?

A case of this kind, and they are of frequent occurrence, was reported in the London newspapers of September 6. While standing on the pavement outside the London Hippodrome two soldiers fell off the kerb as a 'bus was drawing up. One of the men, William Albert Dobson, a driver in the Canadian Field Artillery, was trapped between the 'bus-wheel and the kerb. He was taken to a military hospital in Endell Street, and was examined by a lady doctor, who reported that he was "beastly drunk." She allowed a constable to take him to the police station, where the police doctor certified him drunk and fit to be detained. Dobson was charged with drunkenness and placed in a cell, where the next day he complained of severe internal pains, and was transferred to King George's Hospital. An operation was performed, but death took place from peritonitis set up by squeezing between a wheel and a kerb. A verdict of "Accidental death" was returned, the 'bus-driver being exonerated from all blame. No rider imparting blame to the two doctors who gave the false diagnosis that the man's condition was due to drink was added.

When the smallest quantity of any alcoholic drink recently taken will unavoidably impart its odour to the breath, can anything be more irrational than to conclude that because the odour of alcohol is detectable in the breath of a comatose patient he is necessarily suffering from alcoholic coma, when it may be due to stunning or other injury by falling, or many other causes?—Yours faithfully, WHERE ARE THE CORONERS?

